# The Parkinson's Disease DNA Variant Browser

**DOI:** 10.1002/mds.28488

**Published:** 2021-01-26

**Authors:** Jonggeol J. Kim, Mary B. Makarious, Sara Bandres‐Ciga, Jesse Raphael Gibbs, Jinhui Ding, Dena G. Hernandez, Janet Brooks, Francis P. Grenn, Hirotaka Iwaki, Andrew B. Singleton, Mike A. Nalls, Cornelis Blauwendraat, International Parkinson's Disease Genomics Consortium (IPDGC)

**Affiliations:** ^1^ Molecular Genetics Section, Laboratory of Neurogenetics, National Institute on Aging National Institutes of Health Bethesda Maryland USA; ^2^ Preventive Neurology Unit, Wolfson Institute of Preventive Medicine Queen Mary University of London London UK; ^3^ Computational Biology Core, Laboratory of Neurogenetics National Institute on Aging Bethesda Maryland USA; ^4^ Data Tecnica International Glen Echo Maryland USA

**Keywords:** Parkinson's disease, sequencing, data browser, genetics

## Abstract

**Background:**

Parkinson's disease (PD) is a genetically complex neurodegenerative disease with ~20 genes known to contain mutations that cause PD or atypical parkinsonism. Large‐scale next‐generation sequencing projects have revolutionized genomics research. Applying these data to PD, many genes have been reported to contain putative disease‐causing mutations. In most instances, however, the results remain quite limited and rather preliminary. Our aim was to assist researchers on their search for PD‐risk genes and variant candidates with an easily accessible and open summary‐level genomic data browser for the PD research community.

**Methods:**

Sequencing and imputed genotype data were obtained from multiple sources and harmonized and aggregated.

**Results:**

In total we included a total of 102,127 participants, including 28,453 PD cases, 1650 proxy cases, and 72,024 controls.

**Conclusions:**

We present here the Parkinson's Disease Sequencing Browser: a Shiny‐based web application that presents comprehensive summary‐level frequency data from multiple large‐scale genotyping and sequencing projects https://pdgenetics.shinyapps.io/VariantBrowser/. Published © 2021 This article is a U.S. Government work and is in the public domain in the USA. *Movement Disorders* published by Wiley Periodicals LLC on behalf of International Parkinson and Movement Disorder Society.

Parkinson's disease (PD) is a neurodegenerative disease hallmarked by dopaminergic neuron degradation and Lewy‐body inclusions in the brain. The exact molecular mechanisms underlying PD remain largely unknown, but the disease is influenced by age, environmental, and complex genetic factors. Putative deleterious and highly functional variants in more than 20 genes and 90 common genetic risk variants have been associated with PD or atypical parkinsonism. However, the population risk of known mutations and risk loci only represents a fraction of the known detectable heritable component of disease, suggesting that additional genetic influence is yet to be identified.^1^, ^2^ Most genes associated with PD have been discovered through linkage mapping studies in large family studies, such as *SNCA* ^3^ and *LRRK2*.^4^, ^5^, ^6^ Some studies contain large sequencing cohort validation analyses such as the one nominating *VPS13C*.^7^ The majority of the recent studies that nominate potential PD genes lack replication of results. Current research aggregation resources such as ClinVar are useful for searching known pathogenic variants, but the information presented often misses the context behind the clinical interpretation and lacks large case–control frequency information. Although other resources such as MDSGene (https://www.mdsgene.org/) provide in‐depth genotype–phenotype information, however, they lack large study case–control frequencies.^8^

Next‐generation sequencing has produced petabytes of genomic data and has transformed genomic medicine. However, databases housing these data, such as gnomAD^9^ and BRAVO variant browser,^10^ do not contain disease‐specific data (yet), and there is a need for accessible resources that specifically include allele frequencies per disease group. Here, we aggregated multiple genomic data sets based on PD cases and controls and created an exonic summary data user‐friendly browser, https://pdgenetics.shinyapps.io/VariantBrowser/.

## 1 Methods

### 1.1 Data Aggregation

**FIG. 1:**
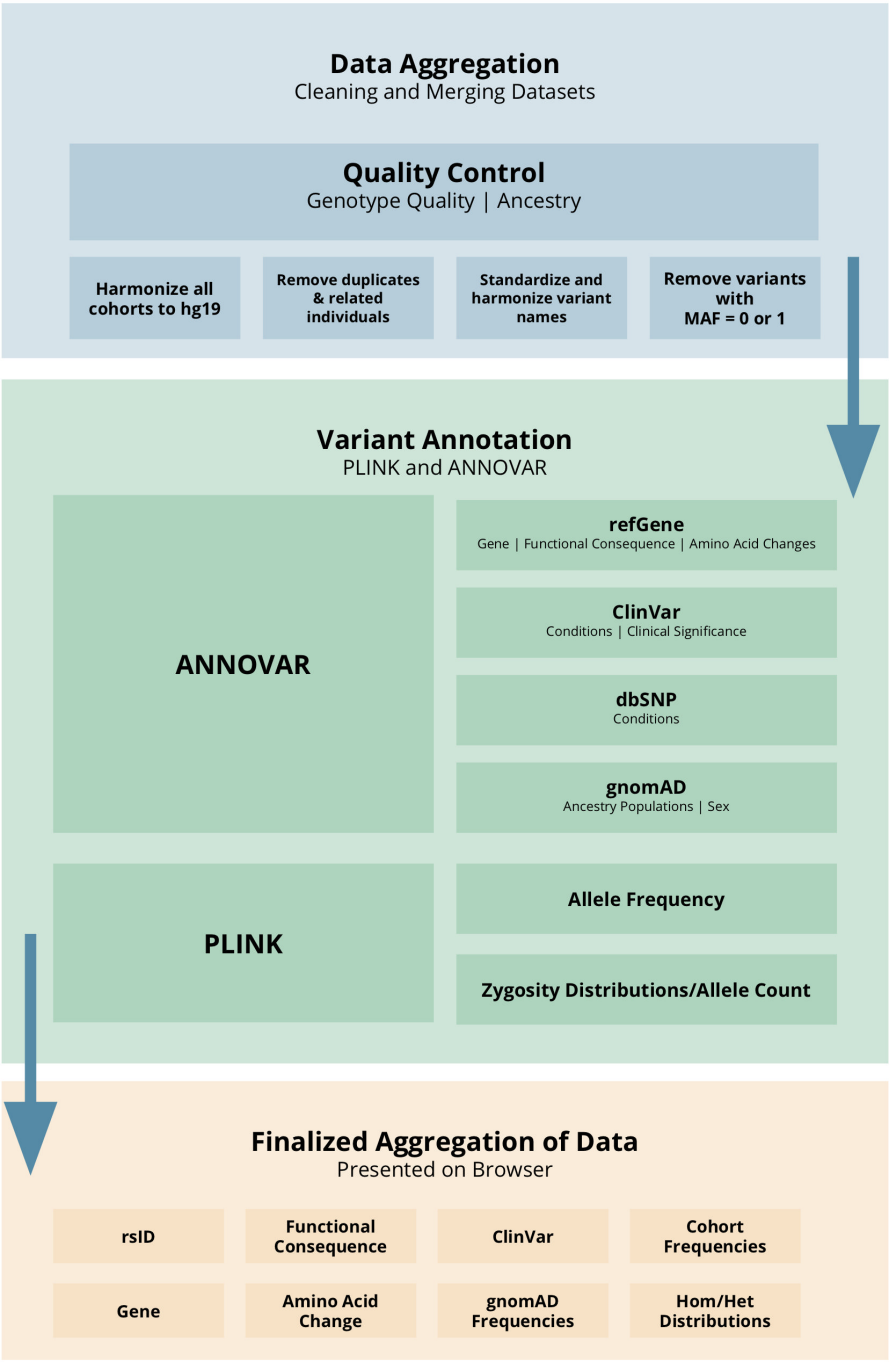
Parkinson's Disease Sequencing Browser processing pipeline flowchart. [Color figure can be viewed at wileyonlinelibrary.com]

We collected sequencing data from multiple different sources (Table 1). The PD Genome Project includes publicly available whole‐genome sequencing data from AMP‐PD (https://amp-pd.org/) and other sources. The International Parkinson's Disease Genomics Consortium (IPDGC) cohort from Parkinson's Disease Genetics Sequencing Consortium (PDGSC) data was downloaded in November 2019 and was processed using a previously described pipeline, https://github.com/ipdgc/pdgsc. The IPDGC resequencing project is a resequencing data set that includes a large number of monogenic genes (*ATP13A2, FBXO7, GBA, LRRK2, MAPT, PARK7* [DJ‐1], *PINK1, PLA2G6, PRKN, SNCA*, and *VPS35*) and genome‐wide association study (GWAS) loci regions from a previous PD GWAS.^11^ The IPDGC genotype data were processed using a previously described quality control pipeline that has been previously described here, https://github.com/neurogenetics/GWAS-pipeline.^1^, ^12^ It was imputed using the Haplotype Reference Consortium Panel and filtered with the estimated r2 (RSQ) threshold of 0.8. UK Biobank (UKB) exome data (field 23160, “Population‐level FE variants, PLINK format”) were downloaded in May 2019.^13^ The PD status of the UKB participants was based on UKB field number 42033, “Source of Parkinson's disease report,” which determined the PD status on 3 criteria: self‐report, hospital admission, and death registries. UKB proxy cases were defined as participants with no PD but with a parent with PD based on UKB field numbers 20107 and 20110, “Illnesses of father” and “Illnesses of mother.” Additional quality control was done to remove participants without case–control status and mean depth of less than 20. Note that the vast majority of data are from European ancestry. Data were trimmed to exome calling regions identical to those used in gnomAD, specifically bait‐covered regions plus 50 bp upstream and downstream. Before merging, all hg38 data were mapped to hg19 using CrossMap v0.4.0,^14^ and each data set was filtered for relatedness by excluding individuals with PIHAT values > 0.125. After merging, duplicate samples were removed based on either sample ID or PIHAT values > 0.8 using PLINK v1.9^15^ (Fig. S1). The data were merged, and allele count and frequency were generated using PLINK. Merged data were annotated using ANNOVAR^16^ (Fig. 1).

**TABLE 1:** Description of the data cohorts

Study	Data Type	Cases (n)	Controls (n)	Total (n)
PD Genome Project^a^	WGS	2745	4071	6816
IPDGC Exomes	WES	2110	2978	5088
IPDGC Resequencing project	Resequencing data	3073	2136	5209
IPDGC GWAS Cohort	Imputed array data	21,412	23,894	45,306
UK Biobank	WES	114	38,263	40,027
UK Biobank (proxy cases)	WES	1650	NA	NA
TOTAL (excluding proxy cases)		29,454	71,342	100,796

WGS, whole‐genome sequencing; WES, whole‐exome sequencing.

^a^ This includes AMP‐PD release 1 genome data (https://amp‐pd.org/).

### 1.2 Browser Design

The IPDGC Sequencing Browser was designed using the Shiny library under R version 3.6.1. All data present in the browser are nonidentifiable aggregate summary‐level data. The design was inspired by the gnomAD and BRAVO variant browsers, featuring gene‐level information panel and separate variant‐level windows. However, this browser increases the information density presented in a single page format with collapsible and information panels to search results facilitating the user visualization and interpretation. It also contains an integrated tutorial function to guide new users. The browser is an open‐source project, and the code is available on our GitHub platform, https://github.com/kimjonggeolj/ipdgc_exome_browser.

## 2 Results

After quality control, we included a total of 6,126,909 variants from 102,446 participants, specifically 29,454 PD cases, 1650 proxy cases from UKB, and 71,342 controls. Of the 3,581,869 exonic variants (~58%), 2,144,315 (~60%) were nonsynonymous variants, and 1,078,658 (~30%) were synonymous variants (Table S1). As a positive control, we assessed the allele frequency of *LRRK2* p.G2019S (rs34637584). This variant is one of the most common PD genetic factors associated with both familial and sporadic forms of disease.^17^ Our browser shows the minor allele frequency of this variant at 0.007716 for cases, 0.0003201 for controls, and 0.001212 for proxy cases. Zygosity distributions show a similar pattern. Of 23,068 cases and 64,036 controls, there are 354 heterozygous cases and 41 heterozygous controls. There is 1 homozygous carrier case, whereas there are no controls with the same zygosity pattern. An association test (chi square allelic test) without adjusting by any covariate but excluding proxy cases showed an odds ratio of 24.28 (95% CI, 17.57–33.6) and a *P* = 1.76 × 10^−179^, which is in line with previous reports for this variant. Another example is the *SNCA* p.A53T (rs104893877) variant, which was the first pathogenic *SNCA* variant described resulting in autosomal‐dominant PD.^3^ The browser shows 2 cases carrying this variant and no controls. The browser can also be used for autosomal‐recessive disorders, for example, using *PRKN* (PARK2) p.R275W (rs34424986) as an example, one of the most common *PRKN* pathogenic variants.^18^ Six cases were homozygous for this variant, and no controls were identified in a homozygous state. The result showed confidence that the data set could be used to identify or provide evidence for a potential PD causal variant.

## 3 Discussion

Here we present the Parkinson's Disease DNA Variant browser, a public platform for the scientific community that allows rapid querying of specific genes and variants in several large case–control cohorts. Provided with a gene name or gene boundaries, the browser will present the user with summarized information on the variants found within the gene, such as the distribution of variants categorized by their functional consequences. Given a specific variant, the browser will present the user with annotated information on the variant including allele frequency, ClinVar information, and functional consequence. These functionalities can be used, for example, when assessing the frequency of a variant of interest identified in a PD case or within a family. As shown in the results of the *LRRK2* p.G2019S, *PRKN* p.R275W, and *SNCA* p.A53T examples, allele frequency and zygosity distribution can give a researcher an idea of whether the reported risk variant may be enriched in PD cases.

Although we performed extensive quality control to ensure high‐quality information was used, the data presented in the browser have inherent limitations. The data sets merged different sequencing technologies including whole‐genome sequencing, whole‐exome sequencing, resequencing, and imputed array‐based genotyping and were aligned using different genome builds such as hg19 and hg38. This leaves gaps from low‐imputation regions and cross‐mapping failures, although the cross‐mapping introduced less than 0.1% reduction in the total number of variants (Fig. S1). Although no sequencing or genotyping method can guarantee perfect data, imputed data because of its nature may add additional uncertainties regarding its results. Our quality control filters reduce this uncertainty, but nevertheless users should always consult the specific study‐level breakdown of the variant frequency and count. The presented data only include autosomal data and do not contain any variants from sex chromosomes. Furthermore, the data presented only include exonic regions and their immediate flank; thus, it cannot provide information on the majority of the noncoding variants. Although this database contains multiple whole‐exome and genome sequencing data, it may still be difficult to identify significant allele frequency and count differences between very rare variants, especially those of recessive inheritance. Researchers should use the annotated information such as ClinVar significance to critically assess any candidate variants. In addition, we only included a very limited amount of phenotypic data with case–control status, which creates potential bias for age‐related penetrance. In future versions, we aim to include age of onset and other phenotype data if available. Of note, the majority of the data included are from European ancestry. We hope in future versions to increase the diversity of the data. Last, some genes, regions of interest, and structural variation are very complicated to genotype and sequence (including the *GBA* gene because of the high similarities with the pseudogene), and therefore interpretation of these complex regions should be done with caution. Future larger‐scale and targeted studies will hopefully resolve the issue with complex genomic regions.

In summary, we present here an online resource developed for the PD research community to quickly retrieve annotated genomic information on genes and variants in a user‐friendly manner, without any required data science or coding experience. Users can access the browser to get information on reported PD risk factors or supplement their own research with data from a large‐scale data set. We envisage this browser to be the first step toward easily sharing genomic information that will be continuously updated as new data become available.

## Full IPDGC Author List

**United Kingdom:** Alastair J. Noyce (Preventive Neurology Unit, Wolfson Institute of Preventive Medicine, QMUL, London, UK, and Department of Molecular Neuroscience, UCL, London, UK), Rauan Kaiyrzhanov (Department of Molecular Neuroscience, UCL Institute of Neurology, London, UK), Ben Middlehurst (Institute of Translational Medicine, University of Liverpool, Liverpool, UK), Demis A. Kia (UCL Genetics Institute; and Department of Molecular Neuroscience, UCL Institute of Neurology, London, UK), Manuela Tan (Department of Clinical Neuroscience, University College London, London, UK), Henry Houlden (Department of Molecular Neuroscience, UCL Institute of Neurology, London, UK), Catherine Storm (Department of Clinical and Movement Neurosciences, UCL Queen Square Institute of Neurology, London, UK), Huw R. Morris (Department of Clinical Neuroscience, University College London, London, UK), Helene Plun‐Favreau (Department of Molecular Neuroscience, UCL Institute of Neurology, London, UK), Peter Holmans (Biostatistics & Bioinformatics Unit, Institute of Psychological Medicine and Clinical Neuroscience, MRC Centre for Neuropsychiatric Genetics & Genomics, Cardiff, UK), John Hardy (Department of Molecular Neuroscience, UCL Institute of Neurology, London, UK), Daniah Trabzuni (Department of Molecular Neuroscience, UCL Institute of Neurology, London, UK; Department of Genetics, King Faisal Specialist Hospital and Research Centre, Riyadh, Saudi Arabia), John Quinn (Institute of Translational Medicine, University of Liverpool, Liverpool, UK), Vivien Bubb (Institute of Translational Medicine, University of Liverpool, Liverpool, UK), Kin Y Mok (Department of Molecular Neuroscience, UCL Institute of Neurology, London, UK), Kerri J. Kinghorn (Institute of Healthy Ageing, Research Department of Genetics, Evolution and Environment, University College London, London, UK), Nicholas W. Wood (UCL Genetics Institute; and Department of Molecular Neuroscience, UCL Institute of Neurology, London, UK), Patrick Lewis (University of Reading, Reading, UK), Sebastian R. Schreglmann (Department of Molecular Neuroscience, UCL Institute of Neurology, London, UK), Ruth Lovering (University College London, London, UK), Lea R'Bibo (Department of Molecular Neuroscience, UCL Institute of Neurology, London, UK), Claudia Manzoni (University of Reading, Reading, UK), Mie Rizig (Department of Molecular Neuroscience, UCL Institute of Neurology, London, UK), Mina Ryten (Department of Molecular Neuroscience, UCL Institute of Neurology, London, UK), Sebastian Guelfi (Department of Molecular Neuroscience, UCL Institute of Neurology, London, UK), Valentina Escott‐Price (MRC Centre for Neuropsychiatric Genetics and Genomics, Cardiff University School of Medicine, Cardiff, UK), Viorica Chelban (Department of Molecular Neuroscience, UCL Institute of Neurology, London, UK), Thomas Foltynie (UCL Institute of Neurology, London, UK), Nigel Williams (MRC Centre for Neuropsychiatric Genetics and Genomics, Cardiff, UK), Karen E. Morrison (Faculty of Medicine, University of Southampton, UK), Carl Clarke (University of Birmingham, Birmingham, UK, and Sandwell and West Birmingham Hospitals NHS Trust, Birmingham, UK), Kirsten Harvey (UCL School of Pharmacy, UK), Benjamin M. Jacobs (Preventive Neurology Unit, Wolfson Institute of Preventive Medicine, QMUL, London, UK).

**France:** Alexis Brice (Institut du Cerveau et de la Moelle épinière, ICM, Inserm U 1127, CNRS, UMR 7225, Sorbonne Universités, UPMC University Paris, UMR S 1127, AP‐HP, Pitié‐Salpêtrière Hospital, Paris, France), Fabrice Danjou (Institut du Cerveau et de la Moelle épinière, ICM, Inserm U 1127, CNRS, UMR 7225, Sorbonne Universités, UPMC University Paris, UMR S 1127, AP‐HP, Pitié‐Salpêtrière Hospital, Paris, France), Suzanne Lesage (Institut du Cerveau et de la Moelle épinière, ICM, Inserm U 1127, CNRS, UMR 7225, Sorbonne Universités, UPMC University Paris 06, UMR S 1127, AP‐HP, Pitié‐Salpêtrière Hospital, Paris, France), Jean‐Christophe Corvol (Institut du Cerveau et de la Moelle épinière, ICM, Inserm U 1127, CNRS, UMR 7225, Sorbonne Universités, UPMC University Paris, UMR S 1127, Centre d'Investigation Clinique Pitié Neurosciences CIC‐1422, AP‐HP, Pitié‐Salpêtrière Hospital, Paris, France), Maria Martinez (INSERM UMR 1220; and Paul Sabatier University, Toulouse, France),

**Germany:** Claudia Schulte (Department for Neurodegenerative Diseases, Hertie Institute for Clinical Brain Research, University of Tübingen, and DZNE, German Center for Neurodegenerative Diseases, Tübingen, Germany), Kathrin Brockmann (Department for Neurodegenerative Diseases, Hertie Institute for Clinical Brain Research, University of Tübingen, and DZNE, German Center for Neurodegenerative Diseases, Tübingen, Germany), Javier Simón‐Sánchez (Department for Neurodegenerative Diseases, Hertie Institute for Clinical Brain Research, University of Tübingen, and DZNE, German Center for Neurodegenerative Diseases, Tübingen, Germany), Peter Heutink (DZNE, German Center for Neurodegenerative Diseases and Department for Neurodegenerative Diseases, Hertie Institute for Clinical Brain Research, University of Tübingen, Tübingen, Germany), Patrizia Rizzu (DZNE, German Center for Neurodegenerative Diseases), Manu Sharma (Centre for Genetic Epidemiology, Institute for Clinical Epidemiology and Applied Biometry, University of Tubingen, Germany), Thomas Gasser (Department for Neurodegenerative Diseases, Hertie Institute for Clinical Brain Research, and DZNE, German Center for Neurodegenerative Diseases, Tübingen, Germany), Susanne A. Schneider (Department of Neurology, Ludwig‐Maximilians‐University Munich, München, Germany).

**United States:** Mark R. Cookson (Laboratory of Neurogenetics, National Institute on Aging, Bethesda, MD), Sara Bandres‐Ciga (Laboratory of Neurogenetics, National Institute on Aging, Bethesda, MD), Cornelis Blauwendraat (Laboratory of Neurogenetics, National Institute on Aging, Bethesda, MD), David W. Craig (Department of Translational Genomics, Keck School of Medicine, University of Southern California, Los Angeles, CA), Kimberley Billingsley (Laboratory of Neurogenetics, National Institute on Aging, Bethesda, MD), Mary B. Makarious (Laboratory of Neurogenetics, National Institute on Aging, Bethesda, MD), Derek P. Narendra (Inherited Movement Disorders Unit, National Institute of Neurological Disorders and Stroke, Bethesda, MD), Faraz Faghri (Laboratory of Neurogenetics, National Institute on Aging, Bethesda, MD; Department of Computer Science, University of Illinois at Urbana‐Champaign, Urbana, IL), Jesse Raphael Gibbs (Laboratory of Neurogenetics, National Institute on Aging, National Institutes of Health, Bethesda, MD), Dena G. Hernandez (Laboratory of Neurogenetics, National Institute on Aging, Bethesda, MD), Kendall Van Keuren‐Jensen (Neurogenomics Division, TGen, Phoenix, AZ), Joshua M. Shulman (Departments of Neurology, Neuroscience, and Molecular & Human Genetics, Baylor College of Medicine, Houston, TX; Jan and Dan Duncan Neurological Research Institute, Texas Children's Hospital, Houston, TX), Hirotaka Iwaki (Laboratory of Neurogenetics, National Institute on Aging, Bethesda, MD), Hampton L. Leonard (Laboratory of Neurogenetics, National Institute on Aging, Bethesda, MD), Mike A. Nalls (Laboratory of Neurogenetics, National Institute on Aging, Bethesda, MD; CEO/Consultant Data Tecnica International, Glen Echo, MD), Laurie Robak (Baylor College of Medicine, Houston, TX), Jose Bras (Center for Neurodegenerative Science, Van Andel Research Institute, Grand Rapids, MI), Rita Guerreiro (Center for Neurodegenerative Science, Van Andel Research Institute, Grand Rapids, MI), Steven Lubbe (Ken and Ruth Davee Department of Neurology and Simpson Querrey Center for Neurogenetics, Northwestern University Feinberg School of Medicine, Chicago, IL), Steven Finkbeiner (Departments of Neurology and Physiology, University of California, San Francisco; Gladstone Institute of Neurological Disease; Taube/Koret Center for Neurodegenerative Disease Research, San Francisco, CA), Niccolo E. Mencacci (Northwestern University Feinberg School of Medicine, Chicago, IL), Codrin Lungu (National Institutes of Health Division of Clinical Research, NINDS, National Institutes of Health, Bethesda, MD), Andrew B. Singleton (Laboratory of Neurogenetics, National Institute on Aging, Bethesda, MD), Sonja W. Scholz (Neurodegenerative Diseases Research Unit, National Institute of Neurological Disorders and Stroke, Bethesda, MD), Xylena Reed (Laboratory of Neurogenetics, National Institute on Aging, Bethesda, MD). Roy N. Alcalay (Department of Neurology, College of Physicians and Surgeons, Columbia University Medical Center, New York, NY; Taub Institute for Research on Alzheimer's Disease and the Aging Brain, College of Physicians and Surgeons, Columbia University Medical Center, New York, NY), Zbigniew K. Wszolek (Department of Neurology, Mayo Clinic Jacksonville, FL), Ryan J. Uitti (Department of Neurology, Mayo Clinic Jacksonville, FL), Owen A. Ross (Departments of Neuroscience & Clinical Genomics, Mayo Clinic Jacksonville, FL), Francis P. Grenn (Laboratory of Neurogenetics, National Institute on Aging, Bethesda, MD).

**Canada:** Ziv Gan‐Or (Montreal Neurological Institute and Hospital, Department of Neurology & Neurosurgery, Department of Human Genetics, McGill University, Montréal, QC, Canada), Guy A. Rouleau (Montreal Neurological Institute and Hospital, Department of Neurology & Neurosurgery, Department of Human Genetics, McGill University, Montréal, QC, Canada), Lynne Krohn (Montreal Neurological Institute and Hospital, Department of Neurology & Neurosurgery, Department of Human Genetics, McGill University, Montréal, QC, Canada), Kheireddin Mufti (Montreal Neurological Institute and Hospital, Department of Neurology & Neurosurgery, Department of Human Genetics, McGill University, Montréal, QC, Canada),

**The Netherlands:** Jacobus J. van Hilten (Department of Neurology, Leiden University Medical Center, Leiden, The Netherlands), Johan Marinus (Department of Neurology, Leiden University Medical Center, Leiden, The Netherlands).

**Spain:** Astrid D. Adarmes‐Gómez (Instituto de Biomedicina de Sevilla [IBiS], Hospital Universitario Virgen del Rocío/CSIC/Universidad de Sevilla, Seville), Miquel Aguilar (Fundació Docència i Recerca Mútua de Terrassa and Movement Disorders Unit, Department of Neurology, University Hospital Mutua de Terrassa, Terrassa, Barcelona), Ignacio Alvarez (Fundació Docència i Recerca Mútua de Terrassa and Movement Disorders Unit, Department of Neurology, University Hospital Mutua de Terrassa, Terrassa, Barcelona),Victoria Alvarez (Hospital Universitario Central de Asturias, Oviedo), Francisco Javier Barrero (Hospital Universitario San Cecilio de Granada, Universidad de Granada), Jesús Alberto Bergareche Yarza (Instituto de Investigación Sanitaria Biodonostia, San Sebastián), Inmaculada Bernal‐Bernal (Instituto de Biomedicina de Sevilla [IBiS], Hospital Universitario Virgen del Rocío/CSIC/Universidad de Sevilla, Seville), Marta Blazquez (Hospital Universitario Central de Asturias, Oviedo), Marta Bonilla‐Toribio (Instituto de Biomedicina de Sevilla [IBiS], Hospital Universitario Virgen del Rocío/CSIC/Universidad de Sevilla, Seville), Juan A. Botía (Universidad de Murcia, Murcia), María Teresa Boungiorno (Fundació Docència i Recerca Mútua de Terrassa and Movement Disorders Unit, Department of Neurology, University Hospital Mutua de Terrassa, Terrassa, Barcelona), Dolores Buiza‐Rueda (Instituto de Biomedicina de Sevilla [IBiS], Hospital Universitario Virgen del Rocío/CSIC/Universidad de Sevilla, Seville), Ana Cámara (Hospital Clinic de Barcelona), Fátima Carrillo (Instituto de Biomedicina de Sevilla [IBiS], Hospital Universitario Virgen del Rocío/CSIC/Universidad de Sevilla, Seville), Mario Carrión‐Claro (Instituto de Biomedicina de Sevilla [IBiS], Hospital Universitario Virgen del Rocío/CSIC/Universidad de Sevilla, Seville), Debora Cerdan (Hospital General de Segovia, Segovia), Jordi Clarimón (Memory Unit, Department of Neurology, IIB Sant Pau, Hospital de la Santa Creu i Sant Pau, Universitat Autònoma de Barcelona and Centro de Investigación Biomédica en Red en Enfermedades Neurodegenerativas [CIBERNED], Madrid), Yaroslau Compta (Hospital Clinic de Barcelona), Monica Diez‐Fairen (Fundació Docència i Recerca Mútua de Terrassa and Movement Disorders Unit, Department of Neurology, University Hospital Mutua de Terrassa, Terrassa, Barcelona), Oriol Dols‐Icardo (Memory Unit, Department of Neurology, IIB Sant Pau, Hospital de la Santa Creu i Sant Pau, Universitat Autònoma de Barcelona, Barcelona, and Centro de Investigación Biomédica en Red en Enfermedades Neurodegenerativas [CIBERNED], Madrid), Jacinto Duarte (Hospital General de Segovia, Segovia), Raquel Duran (Centro de Investigacion Biomedica, Universidad de Granada, Granada), Francisco Escamilla‐Sevilla (Hospital Universitario Virgen de las Nieves, Instituto de Investigación Biosanitaria de Granada, Granada), Mario Ezquerra (Hospital Clinic de Barcelona), Cici Feliz (Departmento de Neurologia, Instituto de Investigación Sanitaria Fundación Jiménez Díaz, Madrid, Spain), Manel Fernández (Hospital Clinic de Barcelona), Rubén Fernández‐Santiago (Hospital Clinic de Barcelona), Ciara Garcia (Hospital Universitario Central de Asturias, Oviedo), Pedro García‐Ruiz (Instituto de Investigación Sanitaria Fundación Jiménez Díaz, Madrid), Pilar Gómez‐Garre (Instituto de Biomedicina de Sevilla [IBiS], Hospital Universitario Virgen del Rocío/CSIC/Universidad de Sevilla, Seville), Maria Jose Gomez Heredia (Hospital Universitario Virgen de la Victoria, Malaga), Isabel Gonzalez‐Aramburu (Hospital Universitario Marqués de Valdecilla‐IDIVAL, Santander), Ana Gorostidi Pagola (Instituto de Investigación Sanitaria Biodonostia, San Sebastián), Janet Hoenicka (Institut de Recerca Sant Joan de Déu, Barcelona), Jon Infante (Hospital Universitario Marqués de Valdecilla‐IDIVAL and University of Cantabria, Santander, and Centro de Investigación Biomédica en Red en Enfermedades Neurodegenerativas [CIBERNED]), Silvia Jesús (Instituto de Biomedicina de Sevilla [IBiS], Hospital Universitario Virgen del Rocío/CSIC/Universidad de Sevilla, Seville), Adriano Jimenez‐Escrig (Hospital Universitario Ramón y Cajal, Madrid), Jaime Kulisevsky (Movement Disorders Unit, Department of Neurology, IIB Sant Pau, Hospital de la Santa Creu i Sant Pau, Universitat Autònoma de Barcelona, Barcelona, and Centro de Investigación Biomédica en Red en Enfermedades Neurodegenerativas [CIBERNED]), Miguel A. Labrador‐Espinosa (Instituto de Biomedicina de Sevilla [IBiS], Hospital Universitario Virgen del Rocío/CSIC/Universidad de Sevilla, Seville), Jose Luis Lopez‐Sendon (Hospital Universitario Ramón y Cajal, Madrid), Adolfo López de Munain Arregui (Instituto de Investigación Sanitaria Biodonostia, San Sebastián), Daniel Macias (Instituto de Biomedicina de Sevilla [IBiS], Hospital Universitario Virgen del Rocío/CSIC/Universidad de Sevilla, Seville), Irene Martínez Torres (Department of Neurology, Instituto de Investigación Sanitaria La Fe, Hospital Universitario y Politécnico La Fe, Valencia), Juan Marín (Movement Disorders Unit, Department of Neurology, IIB Sant Pau, Hospital de la Santa Creu i Sant Pau, Universitat Autònoma de Barcelona, Barcelona, and Centro de Investigación Biomédica en Red en Enfermedades Neurodegenerativas [CIBERNED]), Maria Jose Marti (Hospital Clinic Barcelona), Juan Carlos Martínez‐Castrillo (Instituto Ramón y Cajal de Investigación Sanitaria, Hospital Universitario Ramón y Cajal, Madrid), Carlota Méndez‐del‐Barrio (Instituto de Biomedicina de Sevilla [IBiS], Hospital Universitario Virgen del Rocío/CSIC/Universidad de Sevilla, Seville), Manuel Menéndez González (Hospital Universitario Central de Asturias, Oviedo), Marina Mata (Department of Neurology, Hospital Universitario Infanta Sofía, Madrid), Adolfo Mínguez (Hospital Universitario Virgen de las Nieves, Granada, Instituto de Investigación Biosanitaria de Granada), Pablo Mir (Instituto de Biomedicina de Sevilla [IBiS], Hospital Universitario Virgen del Rocío/CSIC/Universidad de Sevilla, Seville), Elisabet Mondragon Rezola (Instituto de Investigación Sanitaria Biodonostia, San Sebastián), Esteban Muñoz (Hospital Clinic Barcelona), Javier Pagonabarraga (Movement Disorders Unit, Department of Neurology, IIB Sant Pau, Hospital de la Santa Creu i Sant Pau, Universitat Autònoma de Barcelona, Barcelona, and Centro de Investigación Biomédica en Red en Enfermedades Neurodegenerativas [CIBERNED]), Pau Pastor (Fundació Docència i Recerca Mútua de Terrassa and Movement Disorders Unit, Department of Neurology, University Hospital Mutua de Terrassa, Terrassa, Barcelona), Francisco Perez Errazquin (Hospital Universitario Virgen de la Victoria, Malaga), Teresa Periñán‐Tocino (Instituto de Biomedicina de Sevilla [IBiS], Hospital Universitario Virgen del Rocío/CSIC/Universidad de Sevilla, Seville), Javier Ruiz‐Martínez (Hospital Universitario Donostia, Instituto de Investigación Sanitaria Biodonostia, San Sebastián), Clara Ruz (Centro de Investigacion Biomedica, Universidad de Granada, Granada), Antonio Sanchez Rodriguez (Hospital Universitario Marqués de Valdecilla‐IDIVAL, Santander), María Sierra (Hospital Universitario Marqués de Valdecilla‐IDIVAL, Santander), Esther Suarez‐Sanmartin (Hospital Universitario Central de Asturias, Oviedo), Cesar Tabernero (Hospital General de Segovia, Segovia), Juan Pablo Tartari (Fundació Docència i Recerca Mútua de Terrassa and Movement Disorders Unit, Department of Neurology, University Hospital Mutua de Terrassa, Terrassa, Barcelona), Cristina Tejera‐Parrado (Instituto de Biomedicina de Sevilla [IBiS], Hospital Universitario Virgen del Rocío/CSIC/Universidad de Sevilla, Seville), Eduard Tolosa (Hospital Clinic Barcelona), Francesc Valldeoriola (Hospital Clinic Barcelona), Laura Vargas‐González (Instituto de Biomedicina de Sevilla [IBiS], Hospital Universitario Virgen del Rocío/CSIC/Universidad de Sevilla, Seville), Lydia Vela (Department of Neurology, Hospital Universitario Fundación Alcorcón, Madrid), Francisco Vives (Centro de Investigacion Biomedica, Universidad de Granada, Granada).

**Austria:** Alexander Zimprich (Department of Neurology, Medical University of Vienna, Vienna, Austria).

**Norway:** Lasse Pihlstrom (Department of Neurology, Oslo University Hospital, Oslo, Norway), Mathias Toft (Department of Neurology and Institute of Clinical Medicine, Oslo University Hospital, Oslo, Norway).

**Estonia:** Pille Taba (Department of Neurology and Neurosurgery, University of Tartu, Tartu, Estonia).

**Australia:** Sulev Koks (Centre for Molecular Medicine and Innovative Therapeutics, Murdoch University, Murdoch, Perth, Western Australia; Perron Institute for Neurological and Translational Science, Nedlands, Perth, Western Australia).

**Israel:** Sharon Hassin‐Baer (The Movement Disorders Institute, Department of Neurology and Sagol Neuroscience Center, Chaim Sheba Medical Center, Tel‐Hashomer, Ramat Gan, Israel, Sackler Faculty of Medicine, Tel Aviv University, Tel Aviv, Israel).

**Finland:** Kari Majamaa (Institute of Clinical Medicine, Department of Neurology, University of Oulu, Oulu, Finland; Department of Neurology and Medical Research Center, Oulu University Hospital, Oulu, Finland), Ari Siitonen (Institute of Clinical Medicine, Department of Neurology, University of Oulu, Oulu, Finland; Department of Neurology and Medical Research Center, Oulu University Hospital, Oulu, Finland), PenttiTienari (Clinical Neurosciences, Neurology, University of Helsinki, Helsinki, Finland, Helsinki University Hospital, Helsinki, Finland).

**Nigeria:** Njideka U. Okubadejo (University of Lagos, Lagos State, Nigeria), Oluwadamilola O. Ojo (University of Lagos, Lagos State, Nigeria).

**Kazakhstan:** Rauan Kaiyrzhanov (Department of Molecular Neuroscience, UCL Institute of Neurology, London, UK), Chingiz Shashkin (Kazakh National Medical University named after Asfendiyarov, Almaty, Kazakhstan), Nazira Zharkinbekova (South Kazakhstan Medical Academy, Shymkent, Kazakhstan), Vadim Akhmetzhanov (Astana Medical University, Astana Kazakhstan), Akbota Aitkulova (National Center for Biotechnology, Astana, Kazakhstan; Al‐Farabi Kazakh National University, Almaty Kazakhstan), Elena Zholdybayeva (National Center for Biotechnology, Astana, Kazakhstan), Zharkyn Zharmukhanov (National Center for Biotechnology, Astana, Kazakhstan), Gulnaz Kaishybayeva (Scientific and Practical Center “Institute of Neurology named after Smagul Kaishibayev,” Almaty, Kazakhstan), Altynay Karimova (Scientific and Practical Center “Institute of Neurology named after Smagul Kaishibayev,” Almaty, Kazakhstan), Talgat Khaibullin (Semey Medical University, Semey, Kazakhstan).

**Ireland:** Timothy L. Lynch (The Dublin Neurological Institute at the Mater Misericordiae University Hospital, Dublin, Ireland & School of Medicine and Medical Science, University College Dublin, Dublin, Ireland).

## Financial Disclosures

The participation of Drs. Iwaki and Nalls is supported by a consulting contract between Data Tecnica International LLC and the National Institute on Aging, NIH, Bethesda, MD. Dr. Nalls also consults for Genoom Health, Illumina Inc., the Michael J. Fox Foundation for Parkinson's Research, and University of California Healthcare. The other authors declare no competing interests.

## Author Roles

(1) Research project: A. Conception; B. Organization; C. Execution: All; (2) Statistical Analysis: A. Design; B. Execution: All; (3) Manuscript Preparation: A. Writing of the first draft; B. Review and Critique: All.

J.J.K.: 1A, 1B, 1C, 2A, 2B, 3A, 3B.

M.B.M.: 1C, 2B, 3B.

S.B.‐C.: 1C, 2B, 3B.

J.R.G.: 1C, 2B, 3B.

J.D.: 1C, 2B, 3B.

D.G.H.: 1C, 2B, 3B.

J.B.: 1C, 2B, 3B.

F.P.G.: 1C, 2B, 3B.

H.I.: 1C, 2B, 3B.

A.B.S.: 1A, 1B, 1C, 2A, 2B, 3B.

M.A.N.: 1A, 1B, 1C, 2A, 2B, 3B.

C.B.: 1A, 1B, 1C, 2A, 2B, 3A, 3B.

## Supporting information


**Figure S1.** Sankey diagram of the participant filtering pipeline.

**Table S1.** Summary of functional consequence of across variants. *Only variants with condition “Parkinson Disease” and interpretation “Pathogenic.”

Full IPDGC acknowledgment
