# Supplementary figures and images for: The Parkinson's Disease DNA Variant Browser
Source: Mov Disord. 2021 Jan 26;36(5):1250–8. doi: 10.1002/mds.28488 (PMC8248407; doi:10.1002/mds.28488)

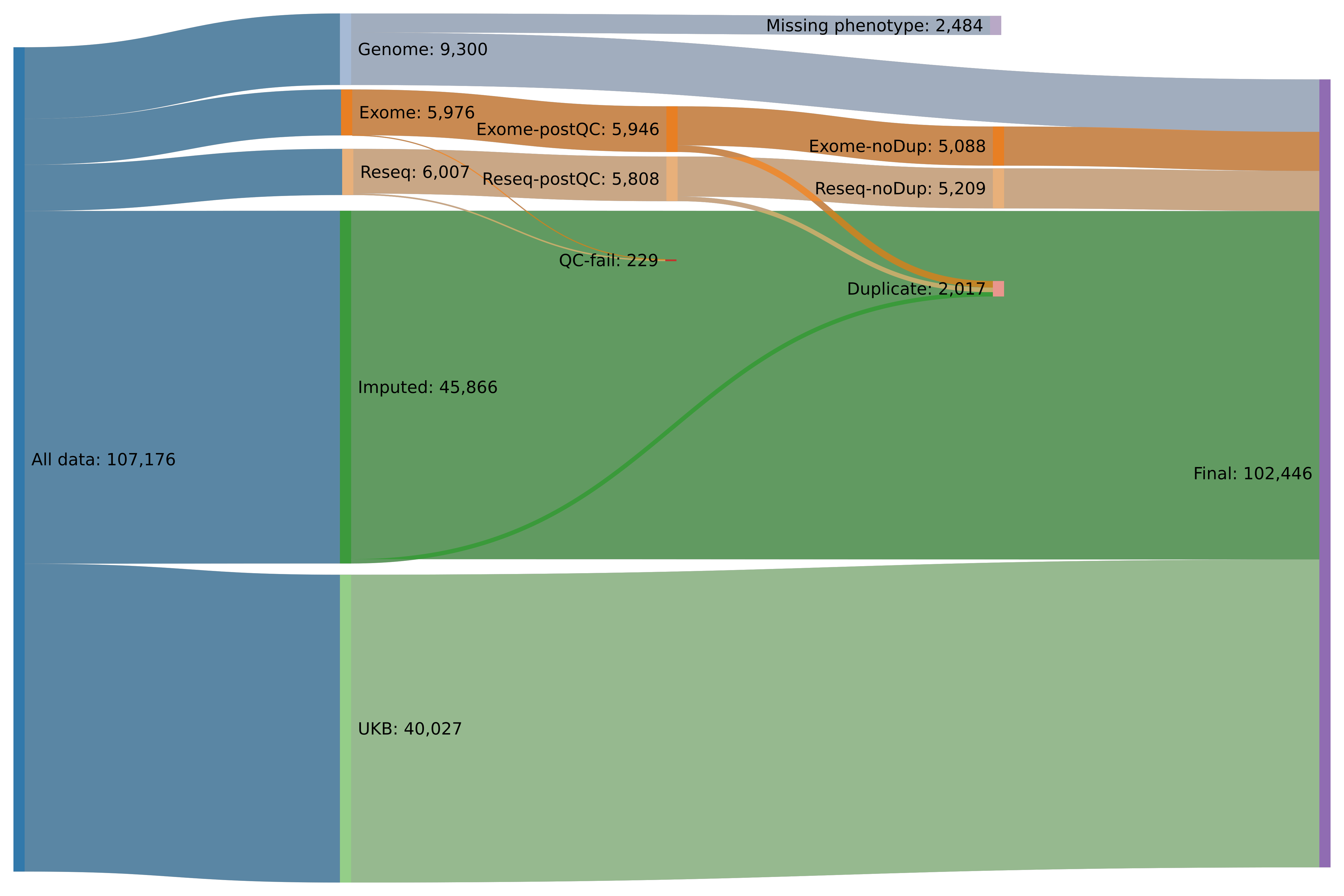

Supplement: Supplementary file 1 — Figure S1. Sankey diagram of the participant filtering pipeline. [file MDS-36-1250-s002.tiff]
