# Supplementary material for: The Parkinson's Disease DNA Variant Browser
Source: Mov Disord. 2021 Jan 26;36(5):1250–8. doi: 10.1002/mds.28488 (PMC8248407; doi:10.1002/mds.28488)

**The Parkinson’s Disease DNA Variant Browser**

Jonggeol J. Kim BSc et al 2020

**Supplementary Table and Figure:**

**Supplementary Table 1:** Summary of functional consequence of across variants. *Only variants with condition “Parkinson Disease” and interpretation “Pathogenic”

|  | **All Variants** | | **Exonic Variants** | | **ClinVar Pathogenic Variants*** | |
| --- | --- | --- | --- | --- | --- | --- |
| **Functional Consequence** | **Counts** | **Percentage** | **Counts** | **Percentage** | **Counts** | **Percentage** |
| **NA** | 2,584,591 | 42.18% | 39,551 | 1.10% | 0 | 0.00% |
| **Unknown** | 91,916 | 1.50% | 91,916 | 2.57% | 0 | 0.00% |
| **Synonymous SNV** | 1,078,658 | 17.61% | 1,078,658 8 | 30.11% | 1 | 3.23% |
| **Nonsynonymous SNV** | 2,144,315 | 35.00% | 2,144,315 | 59.87% | 22 | 70.97% |
| **Stopgain** | 69,780 | 1.14% | 69,780 | 1.95% | 3 | 9.09% |
| **Stoploss** | 3,168 | 0.05% | 3,168 | 0.09% | 0 | 0.00% |
| **Frameshift insertion** | 33,643 | 0.55% | 33,643 | 0.94% | 2 | 6.45% |
| **Frameshift deletion** | 64,177 | 1.05% | 64,177 | 1.79% | 3 | 9.68% |
| **Frameshift substitution** | 88 | 0.00% | 88 | 0.00% | 0 | 0.00% |
| **Nonframeshift insertion** | 19,606 | 0.32% | 19,606 | 0.55% | 0 | 0.00% |
| **Nonframeshift deletion** | 36,936 | 0.60% | 36,936 | 1.03% | 0 | 0.00% |
| **Nonframeshift substitution** | 31 | 0.00% | 31 | 0.00% | 0 | 0.00% |
| **Total** | 6,126,909 | 100.00% | 3,581,869 | 100.00% | 31 | 100.00% |

**Supplementary Figure 1:** Sankey diagram of the participant filtering pipeline


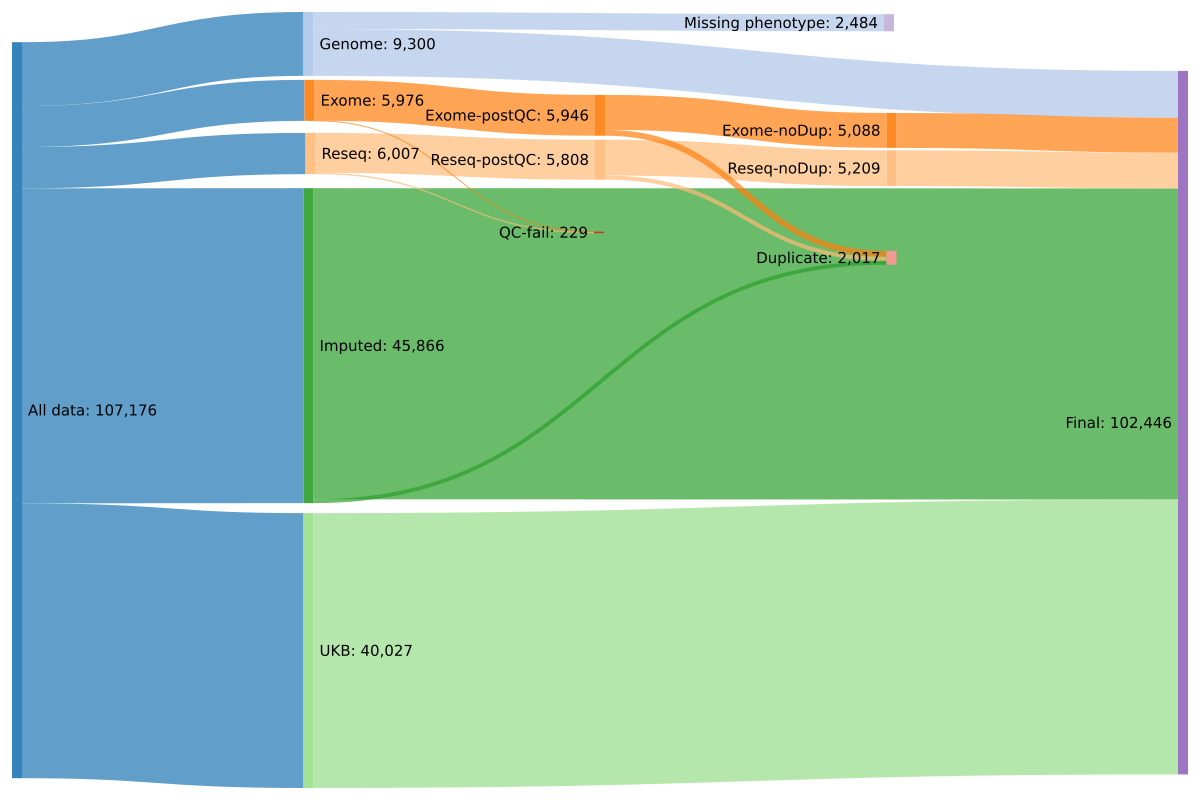

Supplement: Supplementary file 2 — Table S1. Summary of functional consequence of across variants. *Only variants with condition “Parkinson Disease” and interpretation “Pathogenic.” [file MDS-36-1250-s003.docx]
